# Supplementary material for: Intestinal Klebsiella pneumoniae Contributes to Pneumonia by Synthesizing Glutamine in Multiple Myeloma
Source: Cancers (Basel). 2022 Aug 29;14(17):4188. doi: 10.3390/cancers14174188 (PMC9454521; doi:10.3390/cancers14174188)
Supplement: Supplementary file 1 [file cancers-14-04188-s001.zip › cancers-1863229-supplementary.pdf]

Supplementary information

Supplementary Table S1.

List of primers used in this paper.

Gene	Primer	Sequence (5′-3′)
**Primers for intestinal microbiota**		
Total Bacteria*	forward	GTGSTGCAYYGGYTGTCGTCA
	reverse	ACCGTCRTCCMCACCTTCCTC
K. pneumoniae	forward	GCGGACGGGTGAGTAATGTC
	reverse	AGCCGTTACCCCACCTACTA
glnA (qPCR)	forward	AGTGGCAACCCGCTTCAATA
	reverse	GGTTTCGGCATAAAGGTCGC
glnA (PCR)	forward	ATAAGAATGCGGCCGCTAAACTATTTACCACGACGACAATGACCA
	reverse	ATAGTTTAGCGGCCGCATTCTTATGCGGTCGTTCTCTTCCATAC
**Primers for inflammatory factors**		
Mus-GAPDH	forward	TGCACCACCAACTGCTTAG
	reverse	GATGCAGGGATGATGTTC
Mus-IL-1β	forward	ACGGGAAAGACACAGGTAGC
	reverse	AGTTGACGGACCCCAAAAGA
Mus-IL-6	forward	CACGGCCTTCCCTACTTCAC
	reverse	TGCAAGTGCATCATCGTTGT
Mus-TNF-α	forward	ATGGCCTCCCTCTCATCAGT
	reverse	GGCTACAGGCTTGTCACTCG
Homo-GAPDH	forward	TTGCCCTCAACGACCACTTT
	reverse	TGGTCCAGGGGTCTTACTCC
Homo-IL-1β	forward	GTACCTGTCCTGCGTGTTGAA
	reverse	TCTGCTTGAGAGGTGCTGATG
Homo-IL-6	forward	CCAGAGCTGTGCAGATGAGT
	reverse	GTGCCCATGCTACATTTGCC
Homo-TNF-α	forward	AGCCTGTAGCCCATGTTGTAG
	reverse	GAGGAGCACATGGGTGGAG

***:** ‘Total Bacteria’ represents the primer which was used to amply total bacteria [1].

Reference

1. Maeda, H.; Fujimoto, C.; Haruki, Y.; Maeda, T.; Kokeguchi, S.; Petelin, M.; Arai, H.; Tanimoto, I.; Nishimura, F.; Takashiba, S., Quantitative real-time PCR using TaqMan and SYBR Green for Actinobacillus actinomycetemcomitans, Porphyromonas gingivalis, Prevotella intermedia, tetQ gene and total bacteria. *FEMS Immunol Med Microbiol* **2003,** *39* (1), 81-6.

Supplementary Table S2.

The detailed VIP values, FCs, and p-values of differential metabolites.

Metabolites	VIPpred	Fold Change	log2(FC)	t.stat	p-value
4-aminobutyric acid	2.4366	2.3052	1.2049	3.9388	0.0001421
homoserine	2.38232	1.412	0.49774	3.4553	0.0007752
glutamine	2.28564	1.2176	0.28406	3.2652	0.0014483
